# Effectiveness of the Positive Youth Development (PYD) Program on reducing aggression among high school female students

**DOI:** 10.1186/s12905-023-02487-w

**Published:** 2023-06-27

**Authors:** Hassan Zareei mahmoodabadi z, Asieh ebrahimi, Razieh Heydari Sooreshjani

**Affiliations:** Department of Psychology and Educational Sciences, Yazd University, Yazd, Iran

**Keywords:** Positive Youth Development, Aggression, Adolescent, High school student

## Abstract

**Background:**

The positive youth development program highlights the abilities of adolescents and young people. This concept emphasizes that teenagers are capable of making positive changes (such as: flexibility, Responsibility, Identify abilities). This study aimed to determine the effectiveness of the Positive Youth Development (PYD) program on reducing aggression among high school female students in Yazd city. Iran.

**Methods:**

This was a quasi-experimental study, using pretest-posttest design with a control group. In the 2019–2020 academic year, the statistical population comprised all female students in high school first and second years in the city of Yazd, Iran. Using purposive sampling and based on include and exclude criteria (high risk of aggression due to their scores on a screening test and Parental consent to participate in the class) 30 female students were selected. Randomly, they were assigned to an experimental(n = 15) and control groups(n-15). The Buss-Perry Aggression Questionnaire was conducted on students. The experimental group had 8 intervention sessions, whereas the control group received no intervention. The data were subsequently examined using Analysis of Covariance(ANCOVA).

**Results:**

The dependent t-test revealed a significant difference between the pretest-posttest aggression scores in the experimental group, but there was no significant difference in the control group. There was no significant difference in the mean scores of physical aggression, anger, and hostility in the experimental and control groups, but a significant difference was found between the experimental and control groups in the mean verbal aggression scores.

**Conclusion:**

training of positive youth development did not affect on aggression and its three dimensions (physical aggression, anger, and hostility) and only affected adolescent verbal aggression.

## Background

Adolescence is one of the most sensitive stages of life in the development and perfection of physical, mental and social health. For some teenagers, this period has been associated with physical, psychological and social problems, which are a lot of risky behaviors and social harms [1].

Today, in most schools, the aggression of teenagers is observed, and usually the person does not have the strength to control his behavior. In most cases, parents and guardians of schools have conflicts with teenagers, and their actions sometimes cause harassment to the extent that they are expelled from school. If adolescent aggression is not recognized and treated at school, their physical and mental health will be endangered and their academic failure will not be unexpected [2].

According to Delvecchio and Olivery, aggression is distinguished from hostility. Because hostility is an aggressive attitude, but aggression is a visible act of harm [3]. Aggression is a behavior. Boss views aggression as a reaction that exerts deadly stimulation on another person [4]. Social psychologists view aggression as a planned activity intended to cause bodily or mental pain and suffering [5].

Sometimes, the terms anger, hostility, and aggression are used interchangeably. Anger can be considered an emotion, hostility an attitude, and aggression a behavior. According to definitions, anger is an emotion that serves as the foundation for hostility and aggression. Aggression is an observable action carried out with the intent of causing hurt or harm, whereas hostility is an aggressive attitude that motivates an individual to engage in aggressive behavior [6].

The examination of types of aggression by Brotman et al. revealed that aggression might take the form of obvious physical and verbal behavior such as hostile behaviors, shoving, and throwing things. Alternately, it emerges as verbal and hostile threats to commit these acts, such as cursing, shaming, ridiculing, and screaming, which directly damage people [7].

According to studies, lowering high-risk behaviors for prevention alone cannot help teenagers navigate the difficult time of adolescence. Instead, protective elements such as supportive connections in the home and school that aid adolescents’ growth and development should be strengthened [8]. The documents indicate a significant relationship between a teen’s strengths or protective characteristics and a decrease in their engagement in high-risk activities [9]. It is essential to use a well-known framework, the Positive Youth Development Program (PYD), to identify these factors [9].

A recent operational and research framework called the Positive Youth Development Program emphasizes a strengths-based strategy for encouraging positive outcomes for young people [10]. By emphasizing the young people’s flexibility and importance to others and society, this strategy avoids the view that sees kids as problematic and needing reform [11]. Shek et al. define the Positive Youth Development Program as “raising, teaching, and cultivating assets, talents, and potentials associated with adolescent growth and development” [12]. Positive Youth Development, instead of focusing on adolescents’ pathology and issues, stresses their abilities, strengths, and potentials [13] and helps them successfully transition from adolescence [14]. Considerable research on drug abuse, behavioral problems, criminal behavior, academic failure, and adolescent pregnancy has studied the effectiveness of the Positive Youth Development method [15–18, and 19].

In addition, Thebes et al. demonstrated the effectiveness of Positive Youth Development in avoiding non-school-related drug use among adolescents [20, 21]. In a Hong Kong research, a sample of 7151 participants was used to examine the correlations between life satisfaction, positive youth development, and problematic behaviors. The results showed that positive youth development has a positive relationship with life satisfaction, whereas life satisfaction with problematic behaviors has a negative relationship [22].

According to Sharifi et al., [19] in a research on the students of Yazd city, she finds that the atmosphere of the class and family can cause aggression in school. And the aggression of female students is a serious issue for families and school staff in Yazd city. Evidence showed that verbal aggression among female students is more than physical aggression [14, 23]. Friendship groups in school play a decisive role in the occurrence or absence of aggression among female students [19]. Although anger is an emotion, when it leads to aggression, it can lead to injury [3]. Identifying and reacting to this anger is very important and should be reminded to students [3, 16].

The reason for choosing the high school female students was due to the vulnerability of the area and the low income level of the families and the social comparison among the female students. Also, in this region, evidence of conflict between female students in the school environment and also at home with their parents was reported. Although The characteristics of adolescence require that they experience more excitement. But the lack of emotion regulation in teenage girls causes confusion in relationships and also a drop in their education.

Due to the difficulties of adolescence and the likelihood of high-risk behaviors, such as aggression, there is a need in our city for a method to avoid the occurrence of high-risk behaviors among female adolescents, and the Positive Youth Development Program can fill this need. Applying the study’s findings will assist the relevant authorities in developing and putting into practice aggression prevention programs and result in more successful teen-focused initiatives in families, schools, and other support systems. This program keeps teenagers from participating in harmful behaviors like aggression by emphasizing empowerment and creating a safe atmosphere.

Given that little research has been conducted in Iran on the positive youth development program’s effect [23], the present study aims to explore the effect of an educational intervention based on the positive youth development program on reducing student aggression. The researcher is exploring if the Positive Youth Development Program can influence the reduction of aggression among adolescents. So, the hypothesis of the research was that Positive Youth Development Program can reduce aggression in high school female students.

## Method

### Design

This was a quasi-experimental study, using pretest-posttest design with a control group. In the 2019–2020 academic year, the statistical population comprised all female students in high school first and second years in the city of Yazd, Iran. Using purposive sampling and based on including and excluding criteria (high risk of aggression due to their scores on a screening test and Parental consent to participate in the class) 30 female students were selected. Randomly, they were assigned to an experimental(n = 15) and control groups(n-15).

First, an aggression questionnaire was administered to school students, and those students who were diagnosed with aggression were selected, and a semi-structured interview was also used to confirm the accuracy of the collected information. In this interview, the questions were the same for students. For example, how often do you fight with your classmates? Why do you get aggressive? A total of 154 students from four schools were included in the study and 30 students remained based on the entry and exit criteria. The inclusion criteria were Parental consent form, willingness to participate in the research, attending one of the seventh to tenth grades, obtaining a high score in aggression test, and The exclusion criteria was parents’ lack of consent.

The female students in the two experimental and control groups were from 8th to 10th grade, and their average age was about 15 years. They lived with their parents. Parents did not report serious family problems. The average number of children in each family was about 2 children. The income level of the families was medium to high. They lived in the middle area of the city.

Data were analyzed in SPSS 24 using descriptive statistics such as Mean and Standard Deviation and T test& ANCOVA was used to test the research hypothesis. All methods were carried out in accordance with relevant guidelines.

### Intervention

The PYD training program lasted for 2 months and one session of 1.30 h per week. This program was previously implemented by Mazloumi et al. [23] on students. The package was made in consultation with psychology professors of Yazd University and was approved by them. This program has been implemented as a pilot at the school level by researchers. Even this program was coordinated with school administrators and approved for aggression.

A summary of the training sessions is given Table 1.

**Table 1:** Content of sessions

Session	Training content
1	Introduction to the significance of training subjects
2	Improving the awareness, attitude, and skills of students regarding the importance of communication, elements of communication, improving the awareness, attitude, and skills of students regarding the symptoms of anger
3	Improving the awareness, attitude, and skills of students in planning and decision-making, and interpersonal skills
F4	- Improving students’ attitudes and skills regarding positive identity (self-confidence, individual strength) - Improving the attitude and skills of students in recognizing emotions, self-esteem, identity, and setting goals
5	A sense of purpose and a positive view of the individual future
6	Promotion of positive values (compassion, equality, social justice, responsibility, honesty, deterrence)
7	Promoting cultural ability, resistance skills, and conflict resolution methods
8	Improving the components of assertiveness, communication styles, and verbal and non-verbal assertiveness skills, post-test implementation

### Buss-Perry Aggression Questionnaire (BPAQ)

Buss and Perry rewrote the new edition of the aggression questionnaire, previously known as the hostility questionnaire [24]. This questionnaire is a self-report instrument containing 29 statements and four subscales, including physical aggression (PA), verbal aggression (VA), anger (A), and hostility (H). Subjects responded to each of the statements on a 5-point scale from: Like Me To a Great Extent (5), Like Me Somewhat (4), Neither Like Me Nor Not Like Me (3), Not Like Me Somewhat (2), to Not Like Me Not at All (1). The two terms, 9 and 16, are scored inversely. The total score for aggression is obtained by summing the subscales’ scores.

The validity and reliability of the aggression questionnaire are adequate. Retest coefficients for four subscales (with a 9-week interval) range from 0.80 to 0.72, and there is a 0.38–0.49 correlation between the four subscales. The internal validity of the scale was determined using Cronbach’s alpha coefficient, and the findings revealed that the internal consistency of the physical aggression subscale was 0.82, verbal aggression was 0.81, anger was 0.83, and hostility was 0.80 [24].

In a study titled Reliability and Validity of the Buss-Perry Aggression Questionnaire, Samani[25] used factor analysis to extract four behavioral factors: anger, physical and verbal aggression, resentment, and suspiciousness. This questionnaire had a test-retest reliability value equal to 0.78. The strong correlation of the components with the overall score of the questionnaire, the low correlation of other factors, and the alpha coefficient values all show that this questionnaire is adequate and effective for researchers, professionals, and psychologists in Iran [25]. Researchers and professionals may rely on the reliability and validity of the Buss-Perry Aggression Questionnaire.

## Results

The data analysis was done on two levels and through the indicators of descriptive statistics (mean and standard deviation) and inferential statistics.

The descriptive findings of the experimental and control groups in aggression subscales are in Table 2.

**Table 2:** Comparison of the mean and standard deviation of the total score of aggression, physical aggression, verbal aggression, anger, and hostility in the experimental and control groups in the pre-test and post-test stages

Factor	Group	Number	Pre-test		Posttest	
			Mean	Standard deviation	Mean	Standard deviation
Aggression	Experimental	15	99.26	14.08	88.53	16.55
	Control	15	89.93	7.40	86.20	12.76
Physical aggression	Experimental	15	30.66	5.98	26.53	7.28
	Control	15	26.46	5.65	26.33	5.76
Verbal aggression						
	Experimental	15	17.06	3.59	17.73	2.73
	Control	15	15.40	4.27	14.46	3.27
Anger	Experimental	15	23.20	4.21	20.13	4.08
	Control	15	21.86	5.59	20.60	5.05
Hostility	Experimental	15	28.33	5.51	26.60	6.37
	Control	15	26	4.86	26.73	5.11

Table 2 illustrates the decrease in the mean aggression and all its subscales (except for verbal aggression).

The scores’ assumed normality distribution was examined prior to analyzing the aggression data, and the findings supported this assumption’s establishment (p > 0.05). In addition, the homogeneity of regression assumptions was confirmed. Table 3 displays the findings of the covariance analysis of the mean aggression scores of the two experimental and control groups.

**Table 3:** Aggression (general) scores in two groups (ANCOVA)

Variable	Sum of Squares	Degrees of freedom	Mean square	F	Significance level	Eta
Pre-test	1074.81	1	1074.81	5.75	0.020	0.17
Group	53.51	1	53.51	0.28	0.59	0.01
Error	5043.31	27	186.79			

According to Table 3, after adjusting the pre-test scores, there was no significant difference between the experimental and control groups in aggression (general scores) (Eta = 0.01, F, (27,1) = 0.28, Sig > 0.59).

Table 4 illustrates the ANCOVA of the mean physical aggression scores of the two experimental and control groups.

**Table 4:** ANCOVA of post-test scores of physical aggressions in two groups

Variable	Sum of Squares	Degrees of freedom	Mean square	F	Significance level	Eta
Pre-test	767.25	1	767.25	46.88	0.00	0.63
Group	43.69	1	43.69	6.67	0.11	0.09
Error	441.81	27	16.36			

According to Table 5, after adjusting the pre-test scores, there was no significant difference between the experimental and control groups in physical aggression scores (Eta = 0.09, F, (27,1) = 2.67, Sig > 0.11).

Table 5 illustrates ANCOVA of the mean verbal aggression scores of the two experimental and control groups.

**Table 5:** ANCOVA of verbal aggression post-test scores in two groups

Variable	Sum of Squares	Degrees of freedom	Mean square	F	Significance level	Eta
Pre-test	0.005	1	0.005	0.001	0.98	0.0001
Group	76.66	1	76.66	8.12	0.008	0.23
Error	254.66	27	9.43			

According to Table 5, after adjusting the pre-test scores, there was a significant difference between the experimental and control groups in verbal aggression scores (Eta = 0.23, F(27,1) = 8.12, Sig > 0.008).

This means that about 23% of the changes in verbal aggression in students are due to training and this amount is significant.

Table 6 illustrates the ANCOVA of mean anger scores of two experimental and control groups.

**Table 6:** ANCOVA of post-test anger scores in two groups

Variable	Sum of Squares	Degrees of freedom	Mean square	F	Significance level	Eta
Pre-test	119.88	1	119.88	6.86	0.01	0.20
Group	7.71	1	7.71	0.44	0.51	0.01
Error	471.45	27	17.46			

According to Table 6, after adjusting the pre-test scores, there was no significant difference between the experimental and control groups in anger scores (Eta = 0.01, F(27,1) = 0.44, Sig > 0.51).

Table 7 illustrates the ANCOVA of average anger scores of two experimental and control groups.

**Table 7:** ANCOVA of hostility post-test scores in two groups

Variable	Sum of Squares	Degrees of freedom	Mean square	F	Significance level	Eta
Pre-test	132.68	1	132.68	4.45	0.04	0.14
Group	8.76	1	8.76	0.29	0.59	0.01
Error	803.74	27	29.77			

According to Table 7, after adjusting the pre-test scores, there was no significant difference between the two experimental and control groups in hostility scores (Eta = 0.01, F(27,1) = 0.29, Sig > 0.59).

## Discussion

The purpose of the current study was to investigate the effectiveness of the Positive Youth Development Program in reducing adolescent aggression. Based on the findings, positive youth development training had no effect in reducing physical aggression, anger and hostility. But it caused a significant decrease in verbal aggression in children. However, training in Positive Youth Development has helped reduce verbal aggression. Interventions in Positive Youth Development appear to reduce aggressive and violent behavior. However, Roth and Brooks-Gunn have emphasized that further study is necessary to determine the effectiveness of Positive Youth Development treatments in reducing violence [26]. Nonetheless, this study contradicts the findings of Benson and Scales [27].

Verbal aggression is seen within families, in job or educational situations, among students, and even among friends, and causes hostility between people[2, 6]. For students who are subject to verbal or physical violence in the educational environment, in addition to feeling insecure and disliking school, its consequences in their academic life will gradually be revealed in the form of dropping out, and its unpleasant effects will remain with them throughout their lives [28]. Therefore, it is essential to understand the variables contributing to verbal aggression among adolescents to give solutions for its adjustment and resolution. The verbal aggression is important. because it is a precursor to physical violence, lowers students’ self-esteem, and increases the likelihood of disobedience and physical aggression in society [29].

As mentioned, verbal aggression scores have changed in females. The environment of the families of these students was not such that there was a severe problem, but economic problems, class differences can be seen in these families compared to other areas.

The results of Mazloomi et al[23] showed that the income status of families can cause a decrease in students’ self-esteem as well as comparison between classmates. The results of Sharifi et al. [19] also showed that the classroom atmosphere has a great impact on the academic progress and self-esteem of students, and on the other hand, this classroom atmosphere can lead to cooperation or competition between students; This competition can increase violence.

effectiveness of Positive Youth Development treatments in reducing violence [26]. Nonetheless, this study contradicts the findings of Benson and Scales [27].

Beyond the primary social-emotional learning activities, the growth capital strategy to reduce aggression and violence incorporates more basic and systemic reforms. The strategy for producing funding is to support Positive Youth Development and increase the atmosphere for adolescent growth. Due to the considerable changes they bring about in the individual and the environmental system, capital-based behavior modification techniques may have a higher likelihood of success [26]. Within the framework of Positive Youth Development, the family is one of the essential sources of adolescent transformation and growth. Many family responsibilities might either raise or lower the probability of juvenile violence and aggression. Among the most significant causes of juvenile violence in the home are violent and rejecting parents, violence between parents, child abuse and neglect, a chaotic family life, contradicting norms and regulations, and inadequate parental monitoring. These elements may contribute to the development of aggression and violence [30, 31].

## Conclusion

Aggression is a serious issue for families and schools. Investing in this field can be the environment effective for students [31]. Violence can endanger students’ mental health and cause them to fail academically [32]. So, by identifying this violence and using the positive model of youth development, we can emphasize their positive points and take steps to improve their quality level. Families should also know the characteristics of teenagers and get the necessary training in this field [33]. On the other hand, the tendency of teenagers and young people to use virtual space can cause stress and anxiety in them and lead to violence and aggression [34, 35].

### Limitation

Most research has some limits and problems, and this study was no exception. In addition, there were few studies and research in the Positive Youth Development Program, and the participants’ parents were not present in the training courses. One of the most important limitations of this research is that it is not possible to generalize the research results to other societies and male.

### Suggestions

Based on this, it is suggested that school counselors should organize seminars on the prevention of high-risk adolescent behaviors, mainly through the Positive Youth Development Program, so that school counselors can take the necessary precautions to avoid high-risk behaviors more effectively. Teaching the Positive Youth Development Program to parents using the Positive Youth Development intervention package is suggested.

## Data Availability

The datasets used and/or analyzed during the current study available from the corresponding author on reasonable request.
